# Level of dietary protein intake affects glucose turnover in endurance-trained men

**DOI:** 10.1186/1550-2783-8-20

**Published:** 2011-11-16

**Authors:** Stefan M Pasiakos, William F Martin, Charu S Sharma, Matthew A Pikosky, Patricia C Gaine, Douglas R Bolster, Brian T Bennett, Nancy R Rodriguez

**Affiliations:** 1 Department of Nutritional Sciences, University of Connecticut, Storrs, CT, USA

## Abstract

**Background:**

To examine the effects of higher-protein diets on endogenous glucose metabolism in healthy, physically active adults, glucose turnover was assessed in five endurance-trained men (age 21.3 ± 0.3 y, VO_2peak _70.6 ± 0.1 mL kg^-1 ^min^-1^) who consumed dietary protein intakes spanning the current dietary reference intakes.

**Findings:**

Using a randomized, crossover design, volunteers consumed 4 week eucaloric diets providing either a low (0.8 g kg^-1 ^d^-1^; LP), moderate (1.8 g kg^-1 ^d^-1^; MP), or high (3.6 g kg^-1 ^d^-1^; HP) level of dietary protein. Glucose turnover (Ra, glucose rate of appearance; and Rd glucose rate of disappearance) was assessed under fasted, resting conditions using primed, constant infusions of [6,6-^2^H_2_] glucose. Glucose Ra and Rd (mg kg^-1 ^min^-1^) were higher for MP (2.8 ± 0.1 and 2.7 ± 0.1) compared to HP (2.4 ± 0.1 and 2.3 ± 0.2, *P *< 0.05) and LP (2.3 ± 0.1 and 2.2 ± 0.1, *P *< 0.01) diets. Glucose levels (mmol/L) were not different (*P *> 0.05) between LP (4.6 ± 0.1), MP (4.8 ± 0.1), and HP (4.7 ± 0.1) diets.

**Conclusions:**

Level of protein consumption influenced resting glucose turnover in endurance athletes in a state of energy balance with a higher rate of turnover noted for a protein intake of 1.8 g kg^-1 ^d^-1^. Findings suggest that consumption of protein in excess of the recommended dietary allowance but within the current acceptable macronutrient distribution range may contribute to the regulation of blood glucose when carbohydrate intake is reduced by serving as a gluconeogenic substrate in endurance-trained men.

## Introduction

Increasing dietary protein at the expense of carbohydrate in either Type 2 diabetics or in overweight adults in response to energy restriction improves insulin sensitivity and glycemic control [1-5]. Studies have shown that protein intake in excess of the current recommended dietary allowance (RDA: 0.8 g kg^-1 ^d^-1^) stabilizes blood glucose and reduces the postprandial insulin response after weight loss [2,3]. The metabolic advantage of a diet which provides dietary protein above the RDA specific to glucose utilization in healthy, physically active adults is unclear [6].

Higher-protein intakes are recommended for physically active adults who routinely participate in endurance exercise [7-9]. To date, no studies have investigated the impact of dietary protein intake on glucose homeostasis in endurance-trained adults. The objective of our study was to examine the effects of consuming dietary protein intakes spanning the current Acceptable Macronutrient Distribution Range (AMDR) on resting glucose turnover in endurance-trained men [10]. We hypothesized that protein availability would influence glucose turnover during a eucaloric state such that glucose rate of appearance (Ra) would be greater when the proportion of energy derived from dietary protein was increased with a simultaneous reduction in carbohydrate consumption.

## Methods

Using a randomized, crossover design, five endurance-trained men (21.3 ± 0.3 y, 179.1 ± 1.6 cm, 70.6 ± 0.1 kg, 8.7 ± 0.4% fat, VO_2peak _70.6 ± 0.1 mL kg^-1 ^min^-1^) were assigned to a diet providing 0.8 (Low Protein; LP), 1.8 (Moderate Protein; MP) or 3.6 (High Protein; HP) grams of protein per kilogram body mass per day for four weeks. Participants crossed over and consumed each of the remaining diets in randomized order following a 2 wk wash out period between each diet intervention. Actual macronutrient composition of the each diet was 48% carbohydrate (5.4 g kg^-1 ^d^-1^), 26% fat, and 26% protein (3.1 g kg^-1 ^d^-1^) for HP, 60% carbohydrate (7.4 g kg^-1 ^d^-1^), 26% fat, and 14% protein (1.8 g kg^-1 ^d^-1^) for MP, and 66% carbohydrate (8.3 g kg^-1 ^d^-1^), 27% fat, and 7% protein (0.9 g kg^-1 ^d^-1^) for LP. Extended details of the diet intervention have been previously reported [8]. Volunteers maintained their normal level of training throughout the study. However, exercise was restricted for 24 h before glucose turnover assessments to minimize the potential influence of previous exercise on study measures.

Glucose turnover was assessed after 3 wks of each 4 wk diet intervention using a 120 min primed, constant infusion of [6,6-^2^H_2_] glucose (17 μmol kg^-1^; 0.2 μmol kg^-1 ^min^-1^; Cambridge Isotope Laboratories, Andover, MA) at 0700 h after an overnight fast (≥ 10 h). Arterialized blood samples were obtained from a dorsal hand vein at baseline, 60, 75, 90, 105 and 120 min to determine glucose turnover, insulin, and glucose concentrations. Plasma enrichment of [6,6-^2^H_2_] glucose was determined in duplicate with a precision of ± 0.2% SD using a Hewlett Packard 5989A GC-MS (Metabolic Solutions Inc, Nashua, NH). Glucose rates of appearance (Ra) and disappearance (Rd) were calculated using a modified version of the Steele equation [11,12].

Plasma insulin and glucose concentrations were determined using a commercial RIA (DSL-1600, Diagnostic Systems Laboratories, Webster, TX) and automated glucose oxidase-peroxidase method (YSI Model 2300, Yellow Springs Instruments, Yellow Springs, OH), respectively.

Baseline participant characteristics and macronutrient data were described using common descriptive statistics. Shapiro-Wilk tests of normality confirmed that plasma glucose, insulin, and glucose turnover data were normally distributed. Repeated measures ANOVA (within-subjects factors, diet: LP vs. MP. vs. HP; and time: time points over infusion protocols) were used to evaluate effects of dietary protein intake on glucose turnover, insulin, and glucose. In cases in which significant main effects (diet or time) or interactions were present, post hoc analyses were conducted by using Bonferroni adjustments to reduce the type I error rate. The alpha level for significance was set at *P *< 0.05. Data were analyzed using SPSS (version 18.0, 2006; SPSS Inc.) and expressed as means ± SEM.

## Results

**Figure 1:**
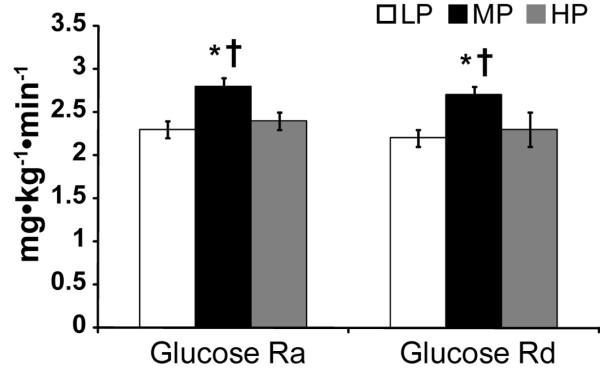
**Glucose turnover**. Glucose rates of appearance (Ra) and disappearance (Rd) for endurance-trained men at rest following 3 wks on the LP, MP and HP diets. Values are presented as mean ± SEM, *n = 5*. * Different from LP, *P *< 0.01. † Different from HP, *P *< 0.05.

Diet main effects (*P *< 0.05) were noted for glucose turnover. Ra (mg kg^-1 ^min^-1^) was greater for MP (2.8 ± 0.1) compared to HP (2.4 ± 0.1, *P *< 0.05) and LP (2.3 ± 0.1, *P *< 0.01) diets **(**Figure 1**)**. Rd (mg kg^-1 ^min^-1^) was also greater for MP (2.7 ± 0.1) than for HP (2.3 ± 0.2, *P *< 0.05) and LP (2.2 ± 0.1, *P *< 0.01) diets (Figure 1). Ra tended to be greater for HP compared to LP (2.4 ± 0.1 vs. 2.3 ± 0.1 for HP and LP respectively, *P *= 0.07). No difference was observed between LP and HP for Rd.

A main effect of diet (*P *< 0.05) was observed for plasma insulin, as mean insulin concentrations (pmol/L) were greater (*P *< 0.01) for LP (49.4 ± 6.4) compared to MP (22.8 ± 2.7) and HP (16.2 ± 0.6) diets. Insulin levels did not change over time (*P *> 0.05). No main effects of time or diet were observed for plasma glucose (mmol/L), as levels remained steady over time and were not different between the LP (4.6 ± 0.1), MP (4.8 ± 0.1), and HP (4.7 ± 0.1) diets (*P *> 0.05). No interactive effects (*P *> 0.05) were observed for plasma glucose and insulin concentrations.

## Discussion

In the present study glucose turnover was greater when protein intake approximated 1.8 g kg^-1 ^d^-1 ^compared to that noted with protein intakes equivalent to the RDA or near the upper limit of the AMDR under fasted, resting conditions in endurance-trained men [10]. To the best of our knowledge, no other studies have examined the influence of dietary protein intake on glucose turnover in endurance-trained men.

Findings from other studies indicate that level of protein intake contributes to glucose homeostasis [1-3,13]. In overweight adult women, a 10 wk, moderate protein (1.5 g kg^-1 ^d^-1^), energy restricted diet stabilized blood glucose and lowered the postprandial insulin response compared to a diet providing protein at 0.8 g kg^-1 ^d^-1 ^[3]. Consistent with the present study, long-term protein intake at 1.9 g kg^-1 ^d^-1 ^increased hepatic glucose output (Ra) compared to that observed when protein intake was 0.7 g kg^-1 ^d^-1 ^[14]. Contrary to our findings, glucose disposal (Rd) was reduced with this level of protein intake. This discrepancy is likely due to differences in study populations and the experimental conditions under which glucose turnover was assessed (i.e., euglycemic hyperinsulinemic clamp vs. normal fasted) [14]. Also, the rigorous dietary control of the present study ensured adequate energy intake for weight maintenance throughout the study thereby minimizing the influence of energy needs on glucose disposal.

Level of dietary protein can affect glucose utilization by: 1) influencing fasted and postprandial insulin secretion; and 2) providing amino acids which serve as substrates and mediators of hepatic gluconeogenesis [4,15]. In the present study, insulin concentrations mirrored dietary carbohydrate intake, which was inversely related to dietary protein intake. Glucose disposal, however, did not correspond to plasma insulin as glucose Rd was greatest for MP compared to LP and HP diets. In addition, there was no effect of dietary protein on plasma glucose concentrations; although we recognize the small sample (n = 5) may have increased the possibility of committing Type II error. Nevertheless, these findings suggest that endogenous glucose utilization might be regulated by modifications in glucose production as well as changes in peripheral insulin sensitivity [4]. Layman et al. reported lower fasting and postprandial blood glucose concentrations with a greater insulin response for overweight women who consumed the RDA for protein compared to 1.5 g kg^-1 ^d^-1^following weight loss [3]. Our findings are consistent with those of Layman and suggest that a lower ratio of carbohydrate to protein in the diet is associated with euglycemia which may be better maintained by endogenous glucose production [3].

The contribution of amino acids to hepatic glucose production as gluconeogenic substrates and through the glucose-alanine cycle is well documented [16-20]. In the present study, glucose Ra was higher for MP vs. LP, suggesting an effect of protein intake on hepatic glucose production. The increased availability of carbohydrate with the consumption of lower dietary protein (i.e., RDA) contributes to higher rates of carbohydrate oxidation and a reduced need for hepatic glucose production. In contrast, when protein intake increased and approached the upper limit of the AMDR, a concomitant increase in protein oxidation should spare carbohydrate use as a fuel thereby reducing the need for endogenous glucose production [8]. Indeed, consistent with this proposed scenario, previously published data from this investigation showed greater carbohydrate and lower protein oxidation for the MP vs. HP diets and increased protein oxidation with increased protein consumption, which is consistent with the higher rate rates of glucose disposal observed for the MP diet [8,21].

Greater carbohydrate uptake and subsequent oxidation likely increased metabolic demand for endogenous hepatic glucose production accounting for the differences noted in glucose Ra in the MP diet. Consistent with our hypothesis, Jungas et al. reported an increase in protein oxidation concomitant with a greater contribution of amino acids to hepatic gluconeogenesis with modest increases in dietary protein [16]. Therefore, we suggest, and our data support, that prolonged consumption of a MP diet, provides a continuous supply of hepatic gluconeogenic precursors that serve to maintain glucose turnover in a fasted state. Our findings further suggest that a ceiling exists for which dietary protein imparts no additional benefit to the regulation of glucose turnover and may, in fact be excessive to the extent where protein is readily oxidized.

In summary, this investigation demonstrated that glucose turnover is influenced by level of dietary protein routinely consumed by a group of endurance-trained men. A novel aspect of this work is that chronic consumption of dietary protein above 1.8 g kg^-1 ^d^-1 ^did not appear to provide any additional benefit towards the regulation of blood glucose. While our findings must be interpreted cautiously due to the specific population studied (i.e., endurance-trained men), small sample size, and state of energy balance (i.e., eucaloric) during which the experimental diets were implemented, the concept is nonetheless intriguing. That is, when carbohydrate intake is within 55-70% of the total energy consumed and adequate to support glycogen replenishment (7.4 g carbohydrate kg^-1 ^d^-1^), dietary protein at a level that exceeds the RDA but is well within the AMDR may contribute to maintenance of blood glucose by serving as gluconeogenic substrate.

## Competing interests

Nancy R. Rodriguez has received honorarium for participation in the speaker bureau for the NCBA and serves on the Protein Advisory Board for the NCBA. Remaining author(s) declare that they have no competing interests.

## Authors' contributions

SMP participated in manuscript preparation, CSS, MAP, PCG, DRB, and BTB participated in data collection, statistical analysis, and manuscript preparation. NRR served as the principal investigator and contributed to study design, data collection, and manuscript preparation. All authors read and approved the final manuscript.

## References

[B1] Gannon MC Nuttall FQ Saeed A Jordan K Hoover H An increase in dietary protein improves the blood glucose response in persons with type 2 diabetes Am J Clin Nutr 2003 78 734 741 14522731 10.1093/ajcn/78.4.734

[B2] Gannon MC Nuttall FQ Effect of a high-protein, low-carbohydrate diet on blood glucose control in people with type 2 diabetes Diabetes 2004 53 2375 2382 10.2337/diabetes.53.9.2375 15331548

[B3] Layman DK Shiue H Sather C Erickson DJ Baum J Increased Dietary Protein Modifies Glucose and Insulin Homeostasis in Adult Women during Weight Loss J Nutr 2003 133 405 410 12566475 10.1093/jn/133.2.405

[B4] Layman DK Baum JI Dietary Protein Impact on Glycemic Control during Weight Loss J Nutr 2004 134 766 779 10.1093/jn/134.4.968S 15051856

[B5] Piatti PM Monti F Fermo I Baruffaldi L Nasser R Santambrogio G Librenti MC Galli-Kienle M Pontiroli AE Pozza G Hypocaloric high-protein diet improves glucose oxidation and spares lean body mass: comparison to hypocaloric high-carbohydrate diet Metabolism 1994 43 1481 1487 10.1016/0026-0495(94)90005-1 7990700

[B6] Brehm BJ D'Alessio DA Benefits of high-protein weight loss diets: enough evidence for practice? Curr Opin Endocrinol Diabetes Obes 2008 15 416 421 10.1097/MED.0b013e328308dc13 18769212

[B7] Bolster DR Pikosky MA Gaine PC Martin W Wolfe RR Tipton KD Maclean D Maresh CM Rodriguez NR Dietary protein intake impacts human skeletal muscle protein fractional synthetic rates after endurance exercise Am J Physiol 2005 289 E678 E683 10.1152/ajpendo.00060.2005 15914508

[B8] Gaine PC Pikosky MA Martin WF Bolster DR Maresh CM Rodriguez NR Level of dietary protein impacts whole body protein turnover in trained males at rest Metabolism 2006 55 501 507 10.1016/j.metabol.2005.10.012 16546481

[B9] Rodriguez NR Di Marco NM Langley S American College of Sports Medicine position stand. Nutrition and athletic performance Med Sci Sports Exerc 2009 41 709 731 10.1249/MSS.0b013e31890eb86 19225360

[B10] Food and Nutrition Board IoM Dietary reference intakes for energy, carbohydrate, fiber, fat, fatty acids, cholesterol, protein, and amino acids 2005 Washington, D.C.: The National Academies Press 10.1016/s0002-8223(02)90346-9 12449285

[B11] Steele R Wall JS De Bodo RC Altszuler N Measurement of size and turnover rate of body glucose pool by the isotope dilution method Am J Physiol 1956 187 15 24 13362583 10.1152/ajplegacy.1956.187.1.15

[B12] Wolfe RR Isotope Tracers in Metabolic Research: Principals and Practice of Kinetic Analysis 2005 Hoboken, NJ.: John Wiley & Sons Inc.

[B13] Braun B Mawson JT Muza SR Dominick SB Brooks GA Horning MA Rock PB Moore LG Mazzeo RS Ezeji-Okoye SC Women at altitude: carbohydrate utilization during exercise at 4,300 m J Appl Physiol 2000 88 246 256 10642387 10.1152/jappl.2000.88.1.246

[B14] Linn T Santosa B Gronemeyer D Aygen S Scholz N Busch M Bretzel RG Effect of long-term dietary protein intake on glucose metabolism in humans Diabetologia 2000 43 1257 1265 10.1007/s001250051521 11079744

[B15] Millward DJ Layman DK Tome D Schaafsma G Protein quality assessment: impact of expanding understanding of protein and amino acid needs for optimal health Am J Clin Nutr 2008 87 1576S 1581S 18469291 10.1093/ajcn/87.5.1576S

[B16] Jungas RL Halperin ML Brosnan JT Quantitative analysis of amino acid oxidation and related gluconeogenesis in humans Physiol Rev 1992 72 419 448 1557428 10.1152/physrev.1992.72.2.419

[B17] Katz J Tayek JA Gluconeogenesis and the Cori cycle in 12-, 20-, and 40-h-fasted humans Am J Physiol 1998 275 E537 E542 9725823 10.1152/ajpendo.1998.275.3.E537

[B18] Krebs M Brehm A Krssak M Anderwald C Bernroider E Nowotny P Roth E Chandramouli V Landau BR Waldhausl W Direct and indirect effects of amino acids on hepatic glucose metabolism in humans Diabetologia 2003 46 917 925 10.1007/s00125-003-1129-1 12819901

[B19] Krebs M Amino acid-dependent modulation of glucose metabolism in humans Eur J Clin Invest 2005 35 351 354 10.1111/j.1365-2362.2005.01506.x 15948894

[B20] Promintzer M Krebs M Effects of dietary protein on glucose homeostasis Curr Opin Clin Nutr Metab Care 2006 9 463 468 10.1097/01.mco.0000232909.84483.a9 16778578

[B21] Vogt C Petrides AS Stimulation of muscle glucose disposal by insulin in humans is a function of the preexisting plasma insulin level Am J Physiol 1995 268 E1031 E1038 7762630 10.1152/ajpendo.1995.268.5.E1031

